# Remarkable consistency of spinal cord microvasculature in highly adapted diving odontocetes

**DOI:** 10.3389/fphys.2022.1011869

**Published:** 2022-11-23

**Authors:** Megan L. Miller, Hillary L. Glandon, Michael S. Tift, D. Ann Pabst, Heather N. Koopman

**Affiliations:** Department of Biology and Marine Biology, University of North Carolina Wilmington, Wilmington, NC, United States

**Keywords:** microvasculature, odontocetes, decompression sickness, spinal cord, nitrogen

## Abstract

Odontocetes are breath-hold divers with a suite of physiological, anatomical, and behavioral adaptations that are highly derived and vastly different from those of their terrestrial counterparts. Because of these adaptations for diving, odontocetes were originally thought to be exempt from the harms of nitrogen gas embolism while diving. However, recent studies have shown that these mammals may alter their dive behavior in response to anthropogenic sound, leading to the potential for nitrogen supersaturation and bubble formation which may cause decompression sickness in the central nervous system (CNS). We examined the degree of interface between blood, gases, and neural tissues in the spinal cord by quantifying its microvascular characteristics in five species of odontocetes (*Tursiops truncatus, Delphinus delphis, Grampus griseus, Kogia breviceps,* and *Mesoplodon europaeus*) and a model terrestrial species (the pig-*Sus scrofa domesticus*) for comparison. This approach allowed us to compare microvascular characteristics (microvascular density, branching, and diameter) at several positions (cervical, thoracic, and lumbar) along the spinal cord from odontocetes that are known to be either deep or shallow divers. We found no significant differences (*p* < 0.05 for all comparisons) in microvessel density (9.30–11.18%), microvessel branching (1.60–2.12 branches/vessel), or microvessel diameter (11.83–16.079 µm) between odontocetes and the pig, or between deep and shallow diving odontocete species. This similarity of spinal cord microvasculature anatomy in several species of odontocetes as compared to the terrestrial mammal is in contrast to the wide array of remarkable physio-anatomical adaptations marine mammals have evolved within their circulatory system to cope with the physiological demands of diving. These results, and other studies on CNS lipids, indicate that the spinal cords of odontocetes do not have specialized features that might serve to protect them from Type II DCS.

## Introduction

The fact that marine mammals do not breathe compressed gas while diving, as human SCUBA divers do, was thought to be part of the explanation as to why they should not suffer from decompression sickness (DCS) (Ridgway, 1986), along with their diving adaptations—see below. DCS begins with supersaturation of the tissues—when the entirety of the dissolved gas tensions and water vapor surpasses the absolute pressure (Moon et al., 1995; Vann et al., 2011). In human diving, this state of supersaturation is caused by the rise of partial pressures of inert gas in tissues that takes place when the gas is inspired and subjected to high hydrostatic pressure (Moon et al., 1995; Vann et al., 2011). Gas bubbles originate from pre-existing gas nuclei in the tissue due to stress-assisted nucleation from musculoskeletal activity (Vann & Clark, 1975; Blatteau et al., 2006). During decompression, if the rate of decrease in ambient pressure is greater than the rate of inert gas washout from the tissue, gas nuclei destabilize (Vann & Clark, 1975; Vann et al., 2011). Nuclei increase in size and become bubbles if the tissue remains supersaturated with gas. These gas bubbles may be stabilized and may overload the capacity of the pulmonary net to filter them out of circulation. As a result, gas bubbles of sufficient size can cause vascular blockages and increased localized pressures (i.e., nerve compression), ultimately contributing to the symptoms associated with DCS. (Moon et al., 1995; Vann et al., 2011). DCS can be classified as either Type I or Type II. Type II DCS, the focus of this study, is more severe and is characterized by injury to the central nervous system, mainly to the spinal cord (Jallul et al., 2007) However, the specific mechanisms of spinal cord injury in Type II DCS remain unknown (Jallul et al., 2007).

Marine mammals have evolved several anatomical and physiological adaptions to cope with the demands and constraints of diving. They have lungs that lack smaller branching respiratory bronchi and stiffened upper airways that receive air from more compressible airways near the alveoli and which collapse at depth (Hooker et al., 2012; Ponganis, 2015). Alveolar collapse may prevent nitrogen gas uptake by the blood beyond a certain critical depth (Kooyman, 1985; Kooyman & Ponganis, 1998; Hooker et al., 2012). These mammals additionally possess a well-developed dive response that functions to conserve oxygen stores, prolong maximum dive times, and limit nitrogen uptake (Scholander, 1963; Kooyman, 1985; Kooyman & Ponganis, 1998; Hooker et al., 2012). Furthermore, blood volume and both hemoglobin and myoglobin concentrations are higher in many marine mammals when compared to terrestrial counterparts and serve as a mechanism to increase total body oxygen stores, which can extend the duration the animals spend utilizing aerobic metabolism during dives (Kooyman et al., 1980; Davis, 2014; Ponganis, 2015). Compared to shallow divers, the total mass of deep divers is mainly devoted to metabolically inexpensive integument, bone, and muscle with a smaller proportion for expensive brain and viscera (Pabst et al., 2016; Rowlands et al., 2021). Additionally, deep divers possess locomotor muscles that contribute to low tissue metabolic rates and high oxygen storage capacity (Kanatous et al., 2002; Velten et al., 2013).

These anatomical and physiological adaptations to diving in marine mammals were thought to prevent DCS. However, recent studies have demonstrated that marine mammals, particularly the deep diving odontocetes, appear to change their dive behavior in response to anthropogenic sound, which could increase their risk of developing DCS (Jepson et al., 2003; Fernández et al., 2005; Cox et al., 2006; Hooker et al., 2009; de Quiros et al., 2012; Hooker et al., 2012). Behavioral responses can include a change in dive profile, staying at depth longer than normal, or remaining at the surface longer than normal (Cox et al., 2006). More specifically, it is hypothesized that repetitive shallow dives, dives that are too shallow for alveolar collapse, are most likely to cause DCS (Zimmer & Tyack, 2007; Fahlman et al., 2009). Sonar activity can cause an avoidance behavior resulting in repetitive dives shallower than the depth of alveolar collapse, so the animal can have a larger horizontal distance traveled away from the sonar disturbance (Zimmer & Tyack, 2007). In shallower dives, addition of metabolic gases increases the end dive nitrogen partial pressure in all tissues, while in deeper and longer dives, it decreased (Fahlman et al., 2009). Additionally, shallower dives keep the circulation exposed to lung gases, therefore, uptake can continue (Fahlman et al., 2009). These physiological responses are thought to be the cause of multiple mass stranding events of beaked whales (Frantzis, 1998; Freitas, 2004; Fernández et al., 2005). One stranding in these species showed lesions coinciding with intravascular and major organ gas emboli in spatial and temporal association with military exercises deploying sonar (Fernández et al., 2005).

Given these observations, it is valuable to assess the potential risk of DCS within marine mammals. The work of McClelland et al. (2012), Gabler et al. (2018), and Gabler-Smith et al. (2020) have demonstrated the potential for gas exchange and nitrogen embolism in some marine mammals in two tissues with high lipid content: blubber (the adipocyte-rich hypodermis covering the body) and acoustic fats (specifically, the fat bodies covering and surrounding the mandibles, associated with sound reception in echolocating odontocetes) (Norris, 1968; Pond, 1998; Lonati et al., 2015). However, there is a lack of research dedicated to the spinal cord, the site of the more severe and potentially fatal Type II form of DCS. The two main factors determining the likelihood of a tissue to absorb nitrogen gas under a given pressure are 1) blood supply and degree of blood/tissue interface, and 2) the solubility of nitrogen gas in that tissue (Bert, 1943; Doolette & Mitchell, 2001). Nitrogen is five times more soluble in lipid than aqueous solutions, making vascularized tissues with high lipid content the most susceptible to nitrogen supersaturation (Weathersby & Homer, 1980). The blood/tissue interface occurs at the level of the microvasculature: the capillaries, microarterioles, and microvenules—the microarterioles and microvenules being the branches of the arterioles and venules that form into the capillary bed (Yuan & Rigor, 2010). It is theorized that the spinal cord is especially susceptible to bubble formation due to its high lipid content, (Palmer et al., 1994; Jallul et al., 2007), although any variation in vascular arrangements in any lipid-rich tissue, such has been shown for blubber (McClelland et al., 2012) will impact nitrogen uptake. However, there are currently no data on nitrogen solubility within the spinal cord lipids, or microvasculature of the spinal cord from any marine mammal species.

Given the wide array of anatomical adaptations to the circulatory system of cetaceans, it would be reasonable to hypothesize that these animals have also evolved microvascular adaptations in their spinal cords. Conversely, the marine mammal dive response concept (Scholander, 1940) indicates that blood flow to the brain and heart is preserved during a dive, at the expense of muscles and other organs; if we expand the brain to include the whole central nervous system, then the spinal cord of cetaceans may not exhibit an altered blood supply. We chose to examine, for the first time, the microvessel characteristics in the spinal cords of members of the odontocetes (toothed whales), and to evaluate whether they display specializations putatively associated with diving, as compared to terrestrial mammals. The specific objectives were to examine 1) microvascular characteristics (microvascular density, microvessel size, and microvessel branching) in the spinal cord of several odontocete species in relation to a related terrestrial mammal (*Sus scrofa domesticus*), 2) microvascular characteristics along the length of the spinal cord, and 3) differences in microvasculature between deep (Risso’s dolphin, pygmy sperm whale, Gervais’ beaked whale) and shallow (bottlenose dolphin and common dolphin) diving odontocetes. We also had the opportunity to compare microvessel characteristics of the spinal cord with previously published data in blubber (McClelland et al., 2012) and acoustic fats (Gabler et al., 2018) for *T. truncatus, G. griseus, K breviceps,* and *M. europaeus.*

## Materials and methods

### Sample collection

**FIGURE 1:**
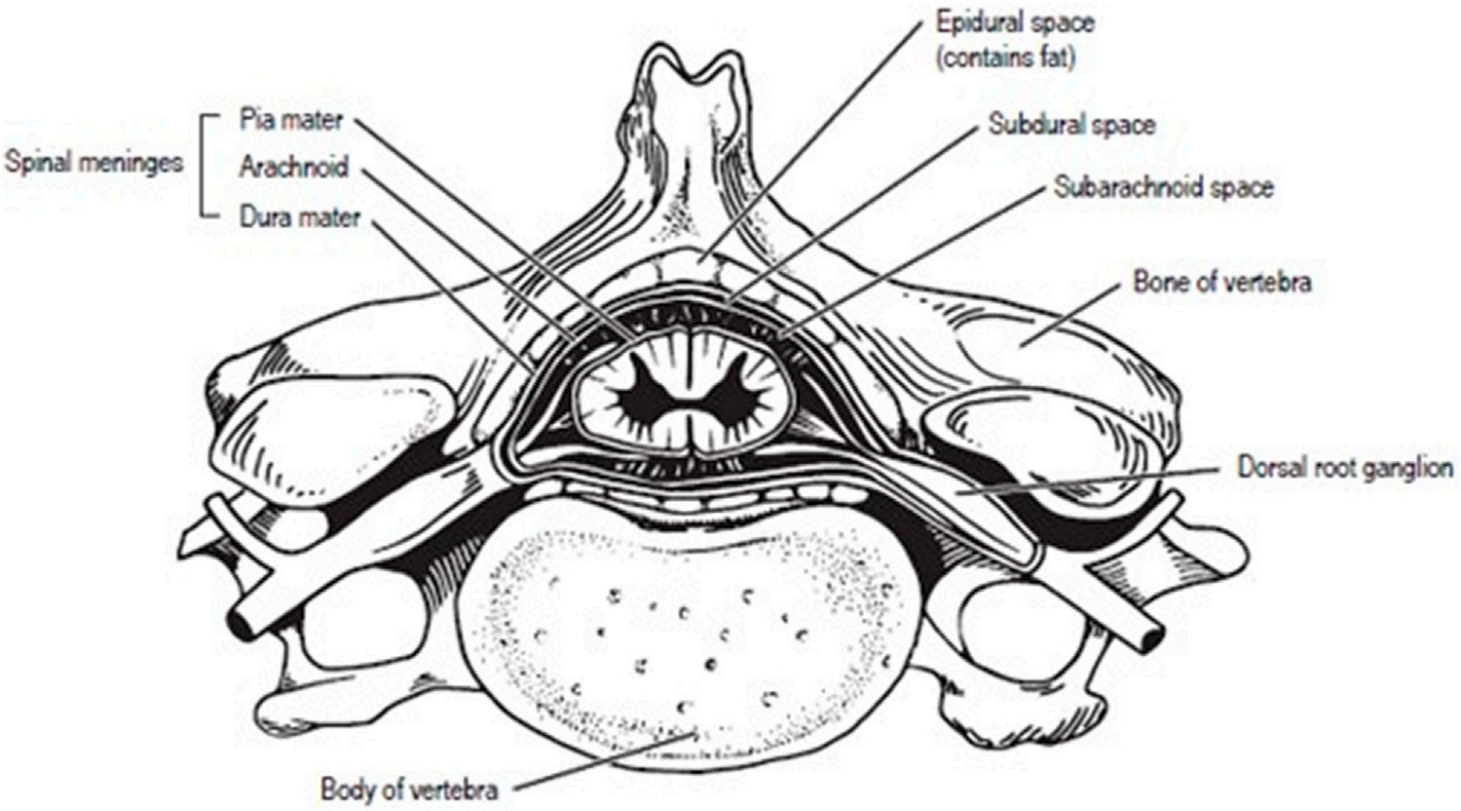
Human Vertebra with labeled spinal cord.

**FIGURE 2:**
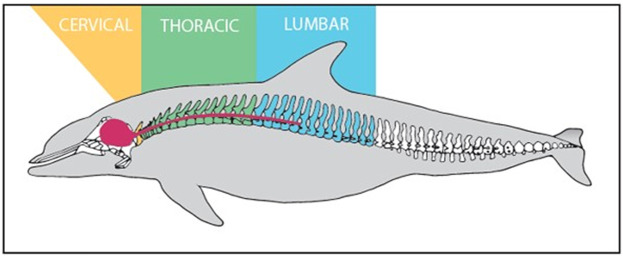
Labeled vertebral column and CNS (Red) of Tursipous truncatus (Rommel, 1990).

Spinal cord samples were collected opportunistically from odontocetes that stranded in good body condition (not emaciated) with a Smithsonian or National Stranding Code ≤2 (a fresh dead carcass) (Geraci & Lounsbury, 2005). Specimens included 10 odontocetes from five different species (two individuals of each species), and two pigs (Table 1—listed by shallow divers, deep divers, and terrestrial species) (Piscitelli et al., 2010). For the odontocetes, necropsies were performed, and the vertebral column was removed, wrapped tightly in plastic bags to avoid desiccation, and stored at −20°C or −80°C until further dissection. The frozen vertebral column was then separated into individual vertebrae using a saw or large knife (Rowlands et al., 2021). Each sample was allowed to partially thaw, and using a scalpel, the spinal cord and meninges were carefully excised from the vertebral canal (Figure 1) and the meninges removed. The spinal cord sample was then cut transversely to collect an approximately 1 cm cross-section, which was placed into a 15 ml cryovial and frozen in a −10°C freezer until further analysis. Samples were variably collected from the cervical, thoracic, and lumbar sections of the spinal cord (Table 1; Figure 2). The exception to this sampling protocol were the pigs. Samples were obtained from a commercial abattoir. Due to health regulations, pig spinal cords were removed from vertebral columns by the commercial processor, and therefore, it was not possible to determine the exact location of samples taken; however, for data analysis purposes, it was assumed pig spinal cord was thoracic. When received, sections of spinal cord were taken equally spaced along the length of the cord.

**TABLE 1:** Spinal cord samples analyzed in this study. Condition code: 1—alive, 2—fresh dead. C—cervical, T—thoracic, L—lumbar.

Sample ID	Species	Dive pattern	Age class	Sex	Length (cm)	Weight (kg)	Condition code	Samples
WAM740	*Tursiops truncatus*	Shallow	Subadult	F	220	128	2	C1-C7, T4, T6, T9, L1, L4
CAHA485	*Tursiops truncatus*	Shallow	Subadult	M	224	121.6	1	T4, T6, T9, L1
IFAW16-032	*Delphinus delphis*	Shallow	Adult	M	226	138.4	2	C7, T1, T7, T9, T12, L2, L3, L5
JPIER010	*Delphinus delphis*	Shallow	Adult	M	220	102	2	C7, T2, T8, T9, T12, L2, L3, L5
CAHA484	*Grampus griseus*	Deep	Adult	F	282	230.4	1	T3, T6, T9, L1
KLC322	*Grampus griseus*	Deep	Subadult	F	247	N/A	2	C1-C7, T4, T6, T9, L1, L4
CER001	*Kogia breviceps*	Deep	Subadult	F	234.5	194	1	C1-C7, T3, T6, T9, L1
KLC334	*Kogia breviceps*	Deep	Adult	M	259	302	2	C1-C7, T4, T6, T9, L1, L3
CAHA476	*Mesoplodon europaeus*	Deep	Adult	F	475	N/A	2	C3-C5, C6, C7, T2, T4, T8, L2, L4, L5
CAHA426	*Mesoplodon europaeus*	Deep	Adult	M	442.5	N/A	2	C1-C7, T4, T6, T9, L1
Pig	*Sus scrofa domesticus*	Terrestrial	N/A	N/A	N/A	N/A	N/A	Thoracic

### Spinal cord sectioning and staining

The frozen, unfixed spinal cord cross-sectional samples from all animals were placed in a Leica Cryocut 1800 (Lecia Biosystems Inc, Buffalo Grove, IL), covered with Optimal Cutting Temperature Compound (Sakura Finetek, Torrence, CA), and allowed to freeze to −27°C. Each spinal cord sample was sectioned to obtain five noncontiguous 20 µm sections, which were placed on gelatin-coated superfrost plus microscope slides (Fisher Scientific, Waltham, MA). All steps following were performed at room temperature (∼20°C). Sections were incubated in BLOXALL Blocking Solution (Vector Laboratories, Burlingame, CA) for 10 min then rinsed in PBS for 5 min. After rinsing, sections were incubated in Normal Horse Serum, 2.5% (Vector Laboratories, Burlingame, CA) for 20 min. The sections were then incubated in CD34 antibody (R&D Systems, Minneapolis, MN) diluted to 1:200 in PBS for 4 h. The sections were then rinsed in PBS for 5 min and incubated in ImmPRESS universal Polymer Reagent (Vector Laboratories, Burlingame, CA) for 30 min. This step was followed by two 5-min rinses in PBS. Afterwards, sections were incubated in ImmPACT DAB EqV (Vector Laboratories, Burlingame, CA) for 5 min. Lastly, sections were rinsed in PBS twice for 5 min and placed under coverslips with trisglycerol.

### Microvascular quantification

Stained sections were viewed with an Olympus BX60 brightfield microscope (Olympus America, Center Valley, PA) and digital images were taken of each section using a Diagnostic Instruments SPOT RT digital camera (Diagnostics Instruments, Sterling Heights, MI). All images were analyzed using ImageJ software (National Institutes of Health, Bethesda, MD). A stereological analysis was utilized by overlaying a grid of 5,000 μm^2^ onto the images. Microvascular density (% microvascularity or area of tissue occupied by microvasculature) was calculated by counting microvessels that hit the overlaying grid in five sections of tissue for each sample to get a total count of at least 100 microvessels (following the methods of (McClelland et al., 2012; Gabler et al., 2018). If the total did not reach 100 with five sections, then more sections were analyzed. To quantify the degree of microvascular branching, five microvessels were randomly selected for each of the five noncontiguous samples. The number of terminal branches per microvessel counted for these 25 microvessels were averaged to determine the number of branches per sample. Microvessel diameter was measured with ImageJ software (Shahlaee et al., 2016). Five microvessels from one image were randomly selected and the diameter was determined by tracing the width of the microvessel from one edge to the other (Shahlaee et al., 2016).

### Statistical analyses

Linear mixed models using repeated measures followed with Type III Analysis of Variance with Satterthwaite’s method (to retrieve *p* values) were run using R version 1.2.5019 (RStudio Inc.—packages lme4 and lmertest) to determine if there were differences in the dependent variables (microvascular density, diameter, and branching) in the three sampling areas of the spinal cords (cervical, thoracic, and lumbar) between the species, and between the different dive behaviors (shallow, deep, terrestrial). The unit of statistical analysis was the average of the dependent variable (density, branching, or diameter) at each location within each individual. The model used to achieve results was as follows: “Parameter (density, branching, or diameter) ∼ Dive Behavior * Location * Dive Behavior:Location + (1/Individual)”. Density, diameter, and branching data were examined for normality using Q-Q plots of residuals *versus* theoretical quantiles and tested for equality of variance using Levene’s test. If there were significant differences in microvascular density, diameter, or branching between the different sampling areas (along the spinal cord) or dive behavior, on each of the variables, post hoc tests were carried out to determine where differences existed. Linear mixed models with random effects (species) of repeated data from multiple animals and multiple tissues were conducted to determine whether there were differences between spinal cord microvasculature and previously published microvasculature data from blubber (McClelland et al., 2012) and acoustic fat bodies (extramandibular fats covering the mandible, and the intramandibular fats in the mandibular fossa) (Gabler et al., 2018). The model used to achieve results was as follows: “Parameter (density, branching, or diameter) ∼ Location + (1/Species). All tests were evaluated for significance at *α* = 0.05.

## Results

### Spinal cord

**FIGURE 3:**
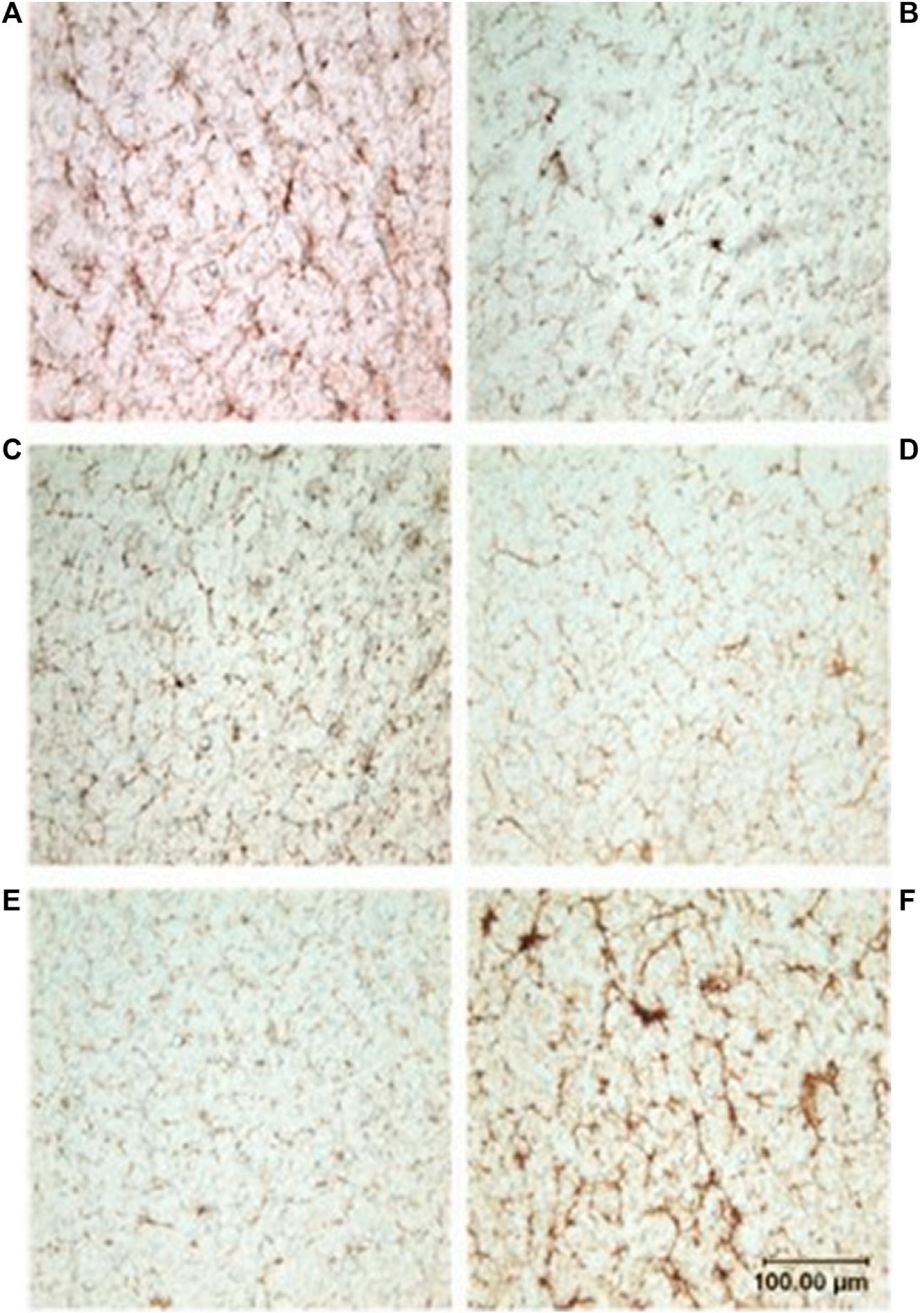
Photomicrographs showing microvasculature of the spinal cord stained with CD34 antibody. Brown-stained lines represent microvessels (10x): **(A)** *D. delphis*, **(B)** *T. truncatus*, **(C)** *G. griseus*, **(D)** *K. breviceps*, **(E)** *M. europaeus*, **(F)** *S. scrofa*.

**FIGURE 4:**
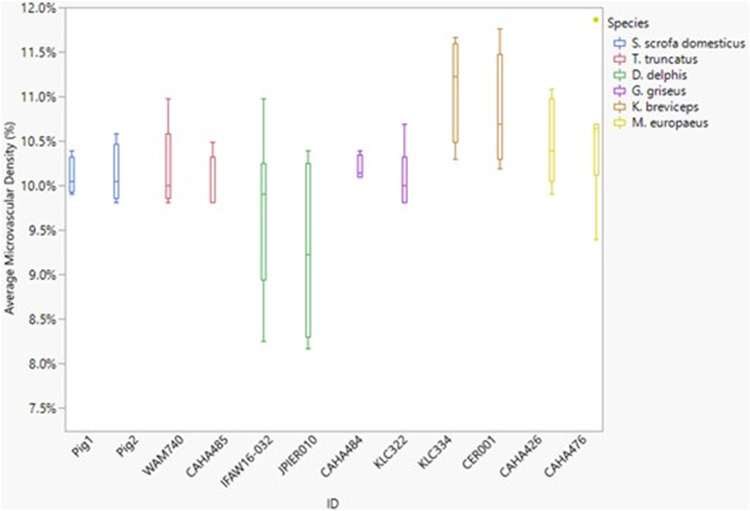
Box and whisker plot of average microvessel density *versus* Animal ID color-coded by species. Horizontal lines from top to bottom: maximum, third quartile, median, first quartile, minimum. Dots represent outliers.

Neither dive behavior (shallow, deep, terrestrial) or spinal cord location (cervical, thoracic, lumbar) had a significant effect on microvascular density (*p* = 0.09 and 0.14, respectively). There was no interaction between dive behavior and spinal cord location (*p* = 0.52). Finally, while *D. delphis* presented the lowest microvascular density and *K. breviceps* had the highest in all sampling locations, the microvascular density varied little between species, ranging from 9.3% to 11% (Figure 3 and Figure 4).

**FIGURE 5:**
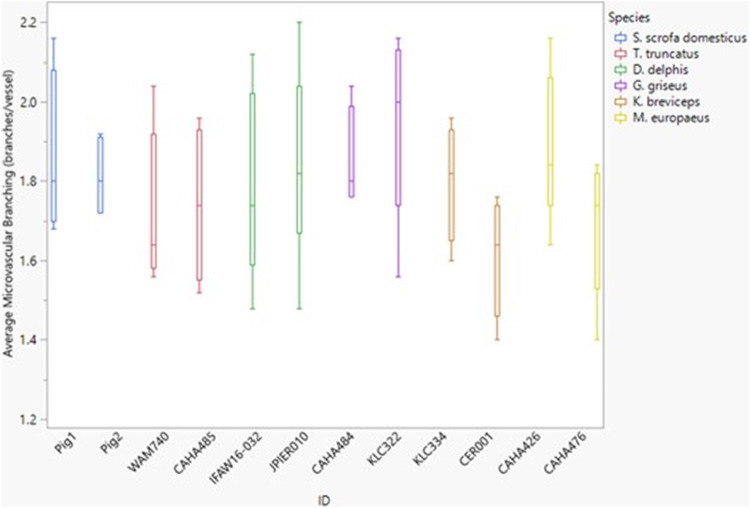
Box and whisker plot of average microvessel branching *versus* Animal ID color-coded by species. Horizontal lines from top to bottom: maximum, third quartile, median, first quartile, minimum.

Between species, microvascular branching was also conserved, ranging from 1.6 to 2.1 branches/microvessel (Figure 3 and Figure 5). Dive behavior and spinal cord location had no significant effect on microvascular branching (*p* = 0.77 and 0.83 respectively) and there was no interaction between dive behavior and spinal cord location (*p* = 0.45).

**FIGURE 6:**
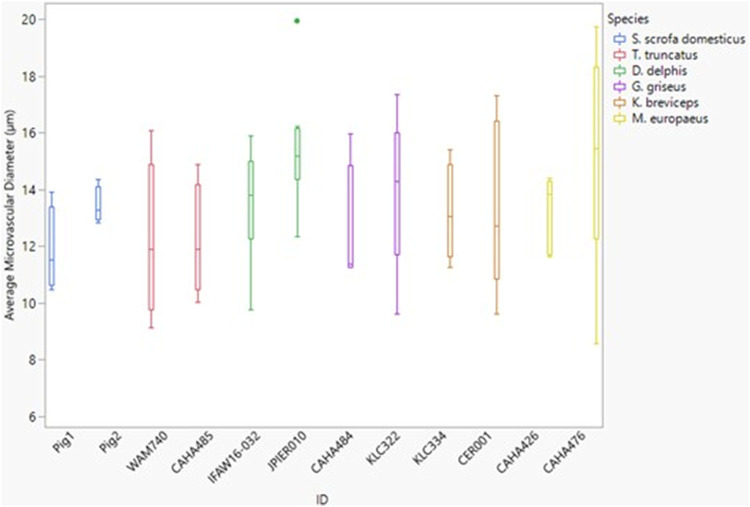
Box and whisker plot of average microvessel diameter *versus* Animal ID color-coded by species. Horizontal lines from top to bottom: maximum, third quartile, median, first quartile, minimum. Dots represent outliers.

The microvessel diameter for all species was between 11 and 15 µm (Figure 6). Neither dive behavior or spinal cord location had a significant effect on microvascular diameter (*p* = 0.93 and 0.25 respectively) and there was no interaction between dive behavior and location (*p* = 0.60).

### Comparisons with other tissues

**FIGURE 7:**
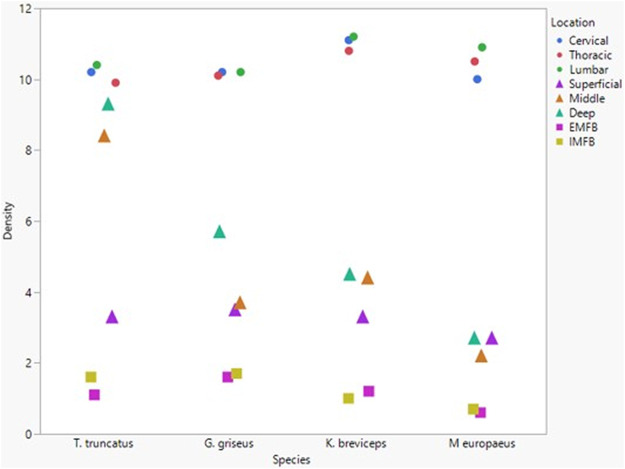
Mean microvascular density in odontocete blubber layers (superficial, middle, deep) (McClelland et al., 2012; triangle), acoustic fats (intramandibular fat bodies—IMFB and extramandibular fat bodies—EMFB) (Gabler et al., 2018; square), and length of spinal cord (cervical, thoracic, lumbar) (circle) tissues.

**FIGURE 8:**
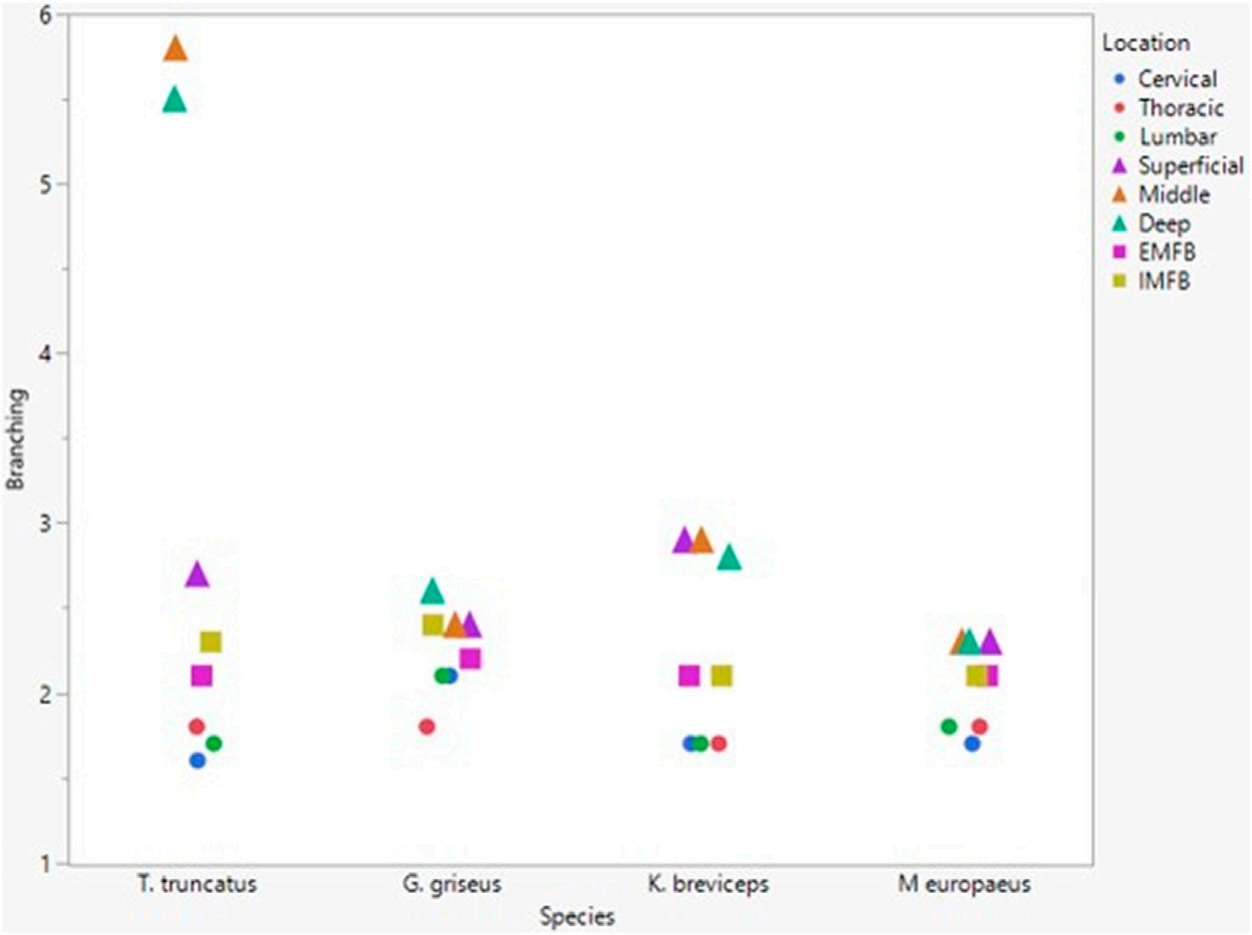
Mean microvascular branching between odontocete blubber layers (superficial, middle, deep) (McClelland et al., 2012; triangle), acoustic fats (intramandibular fat bodies—IMFB and extramandibular fat bodies—EMFB) (Gabler et al., 2018; square), and length of spinal cord (cervical, thoracic, lumbar) (circle) tissues.

**FIGURE 9:**
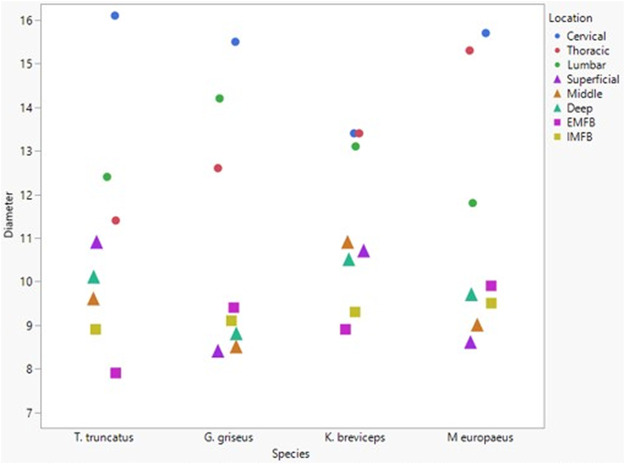
Mean microvascular diameter between odontocete blubber layers (superficial, middle, deep) McClelland et al., 2012; triangle), acoustic fats (intramandibular fat bodies—IMFB and extramandibular fat bodies—EMFB) (Gabler et al., 2018; square), and length of spinal cord (cervical, thoracic, lumbar) (circle) tissues.

All blubber data described here are from McClelland et al. (2012) and all mandibular acoustic fat data are from Gabler et al. (2018). In the acoustic fats, microvascular densities ranged from 0.60 to 1.6% in extramandibular acoustic fats and 0.50 to 1.7% in intramandibular acoustic fats. Within the blubber, microvascular density ranged from 1.60 to 3.30% in superficial (nearest the *epidermis*) blubber and 1.80 to 9.30% in deep (nearest the subdermal connective tissue sheath and muscle) blubber. The spinal cord microvascular density was higher than both blubber and acoustic jaw fats microvascular densities (*p*= < 0.001) (Figure 7). Microvasculature branching ranged from 1.6 to 2.1 branches/vessel in the spinal cord, and 2.1 to 2.9 branches/vessel in the blubber and acoustic fats, except for the middle and deep blubber layers of *T. truncatus* having an average of 5.65 branches/vessel (Figure 8); however, there is still a significant difference (*p* = 0.02) between spinal cord, blubber, and acoustic fats. The microvessel diameter of the spinal cord ranged from 11 to 16 μm, which was larger (*p* = < 0.001) than the microvessel diameter of the blubber and fat bodies (range from 7.90 to 10.9 µm; Figure 9).

## Discussion

This study is the first to measure the microvascular characteristics in the spinal cord for several odontocete species, demonstrating the relative potential (at least in a static sense) for gas exchange between the blood and the tissue of the spinal cord. In all six species studied, both marine and terrestrial, the three microvascular characteristics (microvascular density, branching, and diameter) demonstrated a remarkable overall consistency.

### Microvascular characteristics of odontocete spinal cord

The generally uniform microvascular characteristics between all species, marine and terrestrial, suggests that diving species may not have specialized microvasculature in their spinal cord. Microvessel branching is related to the process of angiogenesis, which is the growth of new blood vessels from pre-existing ones (Baker et al., 2012). Angiogenesis is an intricate process that includes the stimulation of endothelial cells by many different pro-angiogenic factors, including vascular endothelial growth factor (VEGF) and basic fibroblast growth factor (Baker et al., 2012). Expanding blood vessel networks are required to branch frequently to guarantee all tissues receive an adequate blood supply (Ruhrberg et al., 2002). However, these branching networks must obey strict patterning cues to match the architectural designs of the resident tissue (Ruhrberg et al., 2002). The lungs and kidneys best display this pattern demand, in which the juxtaposition of the bloodstream with alveoli or nephric tubules, respectively, is essential for organ function (Abrahamson et al., 1998; Gebb & Shannon, 2000). Thus, it is possible that when blood vessels branch in the spinal cord, they must find pathways around the structures within. Therefore, the similarity in spinal cord microvasculature between species could reflect that the vessels must form around the similar anatomical features of the cord.

**FIGURE 10:**
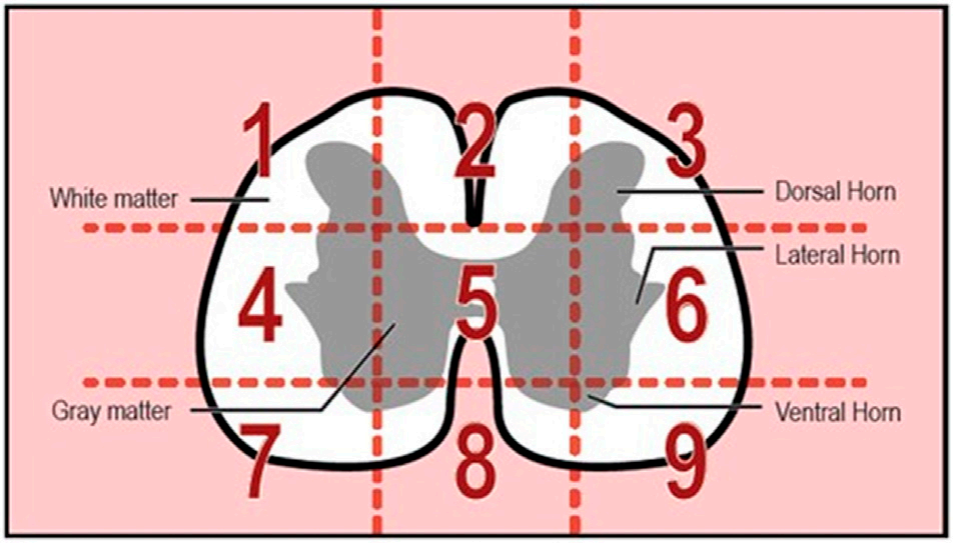
Spinal Cord surface broken into nine sections for image analysis.

**FIGURE 11:**
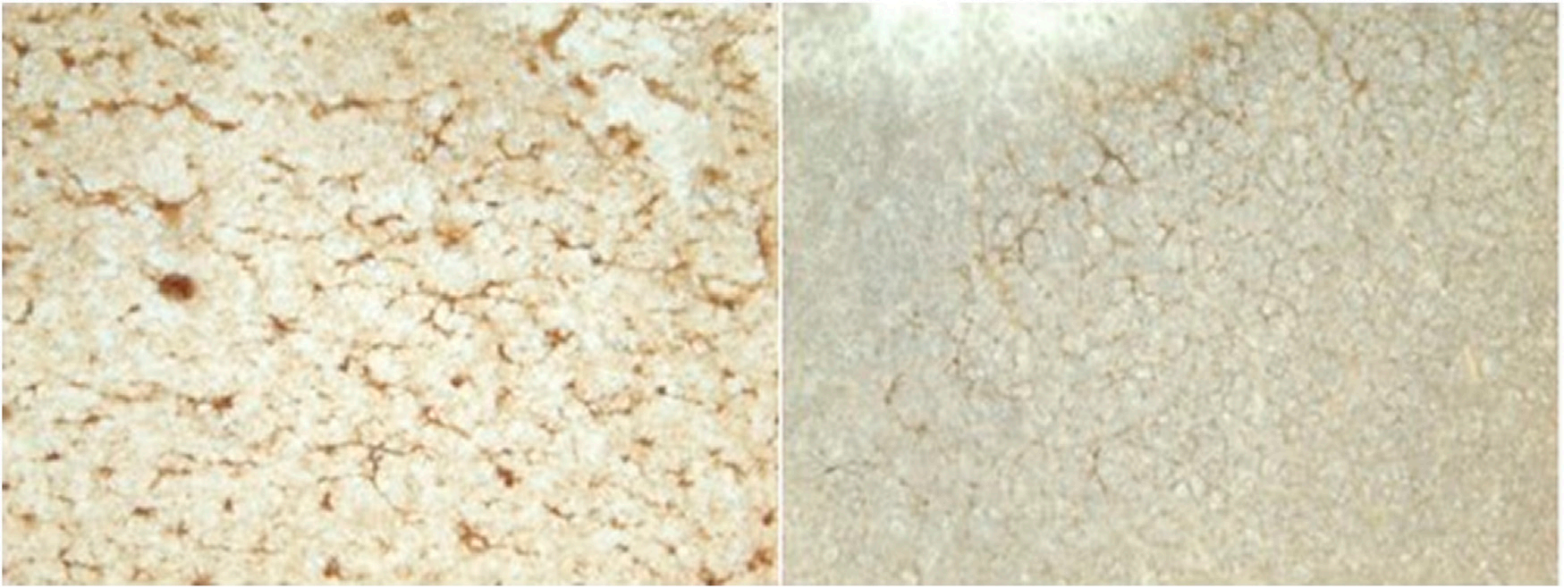
Images of gray matter (Section 5) *versus* white matter (Section 2).

The spinal cord is composed of two general regions: the gray matter and the white matter (Evans & de Lahunta, 2013). The gray matter helps to form the core of the spinal cord and is composed of cell bodies, neurons, glial cells, and a limited amount of myelinated axons (Evans & de Lahunta, 2013). On the other hand, the white matter is situated superficially in the spinal cord and is packed with myelinated axons (Evans & de Lahunta, 2013). One of the key differences between the white and gray matter is that the gray matter of mammals, such as the well-studied dog, has a relatively rich blood supply (Evans & de Lahunta, 2013). Perhaps the differences in the organization of the gray and white matter present developing blood vessels with regionally specific patterning rules. Furthermore, the arrangement could be based on the metabolic requirement of cell bodies and axons (Turnbull, 1971). The white matter microvasculature follows the pattern of the axons—following the direction of the nerve fibers, while the gray matter microvasculature relies on the location of the cell bodies. Images were taken during image acquisition to capture microvessels in both white and grey matter by segregating the spinal cord into nine sections (Figure 10). While all images from areas with the white matter and gray matter were consistent in terms of density and branching, gray matter seems to have microvessels with a greater diameter than vessels of white matter (Figure 11; although we were unable to test whether this difference was significant due to not knowing the exact area of white and gray matter). Greater microvessel diameter in gray matter could correlate to the higher metabolic requirement of the gray matter and blood flow (Marcus et al., 1977). In mammals such as dogs and sheep, the vascularity of the gray matter differs from the white matter since the tissue has a larger metabolic requirement that requires a higher blood flow in this region (Marcus et al., 1977). To accommodate this increase in blood flow, the diameter of vessels increases, allowing lower resistance and constant flow (Marcus et al., 1977).

The average diameter of a red blood cell in odontocetes ranges from 7.3 to 10 µm (Lenfant, 1969) and 5.1 to 8.5 µm in pigs (Scarborough, 1931). Therefore, the microvessel diameters within the spinal cord of both odontocetes and pigs are larger than their average red blood cell diameter, which could be an adaptation related to oxygen and nutrient absorption within the spinal cord. The capillaries are where the exchange of oxygen and nutrients between blood and tissue occurs (Yuan & Rigor, 2010). During dives, marine mammals undergo a well-developed dive response characterized by apnea, bradycardia, and peripheral vasoconstriction (Scholander, 1963; Kooyman, 1985; Hooker et al., 2012; Ponganis, 2015). This results in a redistribution of blood flow so that oxygen sensitive tissues, such as the brain and spinal cord, are adequately perfused with blood, while other tissues experience ischemia and hypoxemia (Zapol et al., 1979; Meir et al., 2009). For example, studies on the Weddell seal (*Leptonychotes weddelli*) show that organs such as the brain and spinal cord maintain or even receive increased blood flow during dives, while organs like the kidneys, spleen, and liver experience decreased blood flow (Zapol et al., 1979). Therefore, the brain and spinal cord receive adequate blood flow during a dive, and the increase in diameter results in lower resistance to further increase the blood flow in these tissues. This allows the tissues like the spinal cord to receive oxygen during times of hypoxia. While terrestrial mammals do not naturally experience hypoxia, it has been shown that hypoxia results in increased blood flow to the brain and spinal cord of terrestrial mammals (Griffiths, 1973; Marcus et al., 1977); mammalian spinal cords may simply be well protected in case of hypoxic events.

The consistency of all microvascular characteristics between species and along the length of the spinal cord parallels a recent study on the lipid composition of spinal cord tissue in similar diving and non-diving species used in this study (Glandon et al., 2021). These authors demonstrated consistency of lipid content and lipid class composition of neural tissues in 12 marine and two terrestrial mammalian species. The five classes of lipids in neural tissue (in terrestrial mammals including dogs and humans and the odontocetes used in Glandon’s study) include sphingomyelin, sulfatides, cerebrosides, phospholipids, and cholesterol. Additionally, the spinal nerve tissue includes triacylglycerol and wax ester/sterol ester (Glandon et al., 2021). Lipid class composition was not significantly different between shallow and deep divers, despite clade-specific differences in the classes of lipids in other tissues, such as blubber (Pond, 1998; Koopman, 2007). Lastly, there was no significant difference in lipid composition across sampling location along the length of the spinal cord (Glandon et al., 2021). Thus, at least from the lipid and microvascular perspectives, the spinal cord appears to be a very conserved tissue across mammals.

### Microvasculature of odontocete tissues

Despite the similarity in microvascular density within the spinal cord, there is significant variation of vascularity between different tissues within odontocetes. The microvascular characteristics have been studied within the acoustic fats and blubber of odontocetes (McClelland et al., 2012; Gabler et al., 2018). A possible explanation for these differences between tissues is that the spinal cord must have higher vascularity to allow for adequate oxygenation of the central nervous system at all times, especially during times of stress on the body (Griffiths, 1973; Marcus et al., 1977; Williams & Davis, 2020).

Preserving blood flow to critical body tissues is supported in diving mammals through the dive response which causes redistribution of blood flow during dives towards oxygen-sensitive tissues (i.e., the central nervous system) and ischemia to hypoxia tolerant tissues (i.e., blubber) (Zapol et al., 1979). The acoustic fats, however, likely sustain perfusion during dives to maintain thermal gradients and acoustic function, though the amount of flow and its consistency is unknown (Houser et al., 2004). Additionally, terrestrial mammals experiencing hyperthermia maintain blood flow to oxygen-sensitive tissues (i.e., the central nervous system) while blood flow to regions of the skeletal muscle and adipose tissue is reduced significantly (Hales et al., 1979). Cerebral autoregulation is not altered while blood flow in other tissues decrease significantly (Ogoh et al., 2013). Thus, increased vascularity of the central nervous system in both marine and terrestrial mammals can aid in autoregulating blood flow to the central nervous system when experiencing environmental stressors. While there are differences in the microvascular density of the various tissues of marine mammals, the branching and diameter are generally consistent between tissues.

### Implications for DCS

Nitrogen gas is approximately five times more soluble in fat than other tissue of the human body (Ikels, 1964). Therefore, since there is more area for gases to diffuse, at most blood/tissue interfaces nitrogen gas will diffuse into the fat, if the partial pressure gradient supports it. This interface occurs at the level of microvasculature (Yuan & Rigor, 2010). Why then have marine mammals not developed their microvasculature as an extra defense against gas embolism?

In diving mammals, the primary determinant of how high the partial pressure of nitrogen rises in blood is determined by the depth of alveolar collapse (Zimmer & Tyack, 2007). The dissolved nitrogen will be distributed throughout perfused tissues based on partial pressure gradients. Bubble nuclei might always be present in diving mammals, but the risk of DCS is dependent on the volume and size of bubbles, resulting in gas supersaturation (Zimmer & Tyack, 2007; Bostrom et al., 2008; Fahlman et al., 2009). The depth of alveolar collapse is dependent on the dead space in the lungs in relation to the total volume of the lung during the dive, thus, different lung volumes will alter the depth of alveolar collapse and produce different equilibrium nitrogen tensions and finally, have an overall influence on the size of any bubbles formed (Zimmer & Tyack, 2007).

It is also proposed that some deep divers, such as the beaked whales, may tolerate sporadic nonlethal bubbles and that normal dive profiles for diving animals show low risk of acute and severe DCS (Zimmer & Tyack, 2007). It has been hypothesized that DCS would be most likely to occur from repetitive shallow dives—dives that are too shallow for alveolar collapse (Zimmer & Tyack, 2007; Fahlman et al., 2009). These cases with limited surface durations and repetitive short dives—correlated to avoidance behavior of acoustic events—are associated with the central circulation and the brain (Frantzis, 1998; Houser et al., 2001; Fernández et al., 2005; Zimmer & Tyack, 2007). This can be seen by looking at the diffusion halftimes of these tissues (Zimmer & Tyack, 2007). The central circulation tissues and brain may exceed their long-term equilibrium tensions during descent which may increase the opportunity for bubble growth at the surface (Zimmer & Tyack, 2007). Despite millions of years of potential selective pressure on the microvasculature and lipids of the cetacean spinal cord, we did not find any evidence of such adaptations for diving in this tissue. We propose that there are physiological limits and trade-offs to the spinal cord, because of the need to deliver blood flow consistently to this oxygen-sensitive tissue, that can override even strong evolutionary pressures associated with diving.

With the lack of microvascular adaptations within the spinal cord, diving vertebrates must avoid gas embolism by minimizing nitrogen uptake and its movement. This can be done by 1) diving with a low diving lung volume, 2) alveolar collapse, and 3) cardiovascular management of systemic blood flow (Fahlman et al., 2021).

## Conclusion

This study is the first to measure the microvascular characteristics in the spinal cord of odontocetes. We found that the spinal cord microvascular characteristics are, overall, consistent between species examined here and along the length of the spinal cord. Moreover, the similarity of odontocete spinal cord microvasculature and that of pig suggests that spinal cord microvasculature is not “adapted” for diving, at least in the animals studied here. Furthermore, the results of this study, combined with the lipid data of Glandon et al. (2021), indicate that many biochemical and anatomical features of the spinal cord are quite conserved when comparing marine and terrestrial mammals.

Despite the variety of adaptations to other areas of the body and patterns of blood flow during dives, it is not known how the risk of Type II spinal cord DCS varies under different dive conditions. The dynamics of blood flow to the spinal cord, as well as nitrogen solubility in the spinal cord are still unknown. Studies to determine the blood flow of the spinal cord during and between dives and the nitrogen solubility in the spinal cord (a goal our lab is working towards) are crucial to fully understanding gas dynamics in these mammals.

## Data Availability

The raw data supporting the conclusion of this article will be made available by the authors, without undue reservation.
